# Neck circumference as an indicator of elevated blood pressure independent from body composition: implications from the China nation health survey (CNHS)

**DOI:** 10.1186/s12872-019-1227-8

**Published:** 2019-11-06

**Authors:** Huijing He, Li Pan, Feng Liu, Jingang Ma, Li Wang, Zhiping Hu, Yajun Li, Guangliang Shan

**Affiliations:** 1 Department of Epidemiology and Statistics, Institute of Basic Medical Sciences, Chinese Academy of Medical, Beijing, 100005 China; 2 Department of Epidemiology and Statistics, School of Basic Medicine, Peking Union Medical College, Beijing, 100005 China; 3 Shaanxi Provincial Center for Disease Control and Prevention, Xi’an, 710054 China

**Keywords:** Neck circumference, Adiposity, Body composition, Blood pressure, Hypertension

## Abstract

**Background:**

The independent association of neck circumference (NC) on elevated blood pressure is still uncertain in adults of China. The aim of this study is to explore such association and investigate the predictive value of NC on hypertension.

**Methods:**

A total of 4279 adults aged 20–80 years participated in the cross-sectional study in 2014. Anthropometric information, NC, body composition indexes such as waist circumference, hip circumference and body fat percentage, and blood pressure were measured. General linear regression model was used to explore the association between NC and blood pressure; Logistic regression models were fitted to calculate the multi-variable adjusted prevalence, and the association of NC with hypertension.

**Results:**

The overall prevalence of hypertension and pre-hypertension were 11.05 and 23.63%, respectively. NC was positively associated with both SBP and DBP (*p* < 0.001). The adjusted prevalence of hypertension increased with elevated NC quartiles in both sexes (*p* for trend < 0.001), from 17.81 to 42.93% in male and 9.72 to 21.31% in female. For male, NC was only associated with hypertension (OR: 1.15, 95% CI: 1.05–1.25) but not with pre-hypertension (OR: 0.97, 95% CI: 0.88–1.06). However, in female, NC was associated with both pre-hypertension and hypertension, the BMI adjusted ORs were 1.15 (1.03–1.28) and 1.24 (1.14–1.34). The sex-specific AUCs for NC predicting hypertension were 0.633 in male and 0.663 in female, similar with AUCs of other body fat indexes.

**Conclusions:**

NC was associated with both pre-HTN and HTN independent from other body composition indexes. NC is a simple and useful anthropometric index to identify elevated blood pressure in Chinese adults.

## Background

Excess adiposity is an important risk factor for cardiometabolic diseases including hypertension, diabetes, dyslipidemia and coronary heart disease [1, 2]. Upper body distribution of fat, especially with increased visceral fat, is more predictive of the metabolic complications of obesity than is the degree of overweight [3]. As an index of upper-body subcutaneous adipose tissue distribution [4], neck circumference (NC) was found associated with cardiometabolic risk factors in populations with diverse ethnic backgrounds [5–8]. Comparing with standard measures such as body mass index, neck circumference may be a convenient and valid alternative measure of obesity and may even be a better marker of metabolic risk [7].

As one of the most important risk factors of cardiovascular disease, hypertension was the world’s leading risk for death [9, 10]. China, as the most populous nation in the world, has nearly 244.5 million (23.2%) hypertension (HTN) patients according to a recent national survey [11]. The prevalence of pre-HTN in adults were as high as 41.3% [11]. There are previous studies reported the association between neck circumference and elevated blood pressure in populations in China, but with inconsistent conclusions. For example, Liang’s study reported a negative association between NC and blood pressure after the adjustments of BMI or waist circumference [4], whereas Zhou and Fan’s studies revealed positive relationships between NC and HTN [6, 12]. Additionally, there is limited data exploring the association between NC and blood pressure covering population in a relatively young age. Therefore, the aim of the present study, is using data derived from the China National Health Survey (CNHS) [13, 14], to explore the relationship between neck circumference and blood pressure among adults aged 20–80 years in China.

## Methods

### Study population

In 2015, we conducted a cross-sectional study in Shaanxi province, northwest China. The criteria for participant recruitment were: 1) aged 20–80 years; 2) local residence for at least 1 year. The exclusion criteria were: 1) woman who is pregnant; 2) soldier in service; 3) disabled individual (who may not be able to complete the whole physical examination) and 4) individual who reported a history of obstructive sleep apnoea. The presented study was part of the China National Health Survey (CNHS). The CNHS used a multi-stage stratified cluster sampling method to select participants. The sampling procedure was described elsewhere [13], which briefly was: in the first stage, the provincial capital and one mid-size city, and two counties including one developed and one undeveloped according to the local GDP were selected; in the second stage, districts were selected from cities, and rural townships were selected from counties; in the next stage, streets or communities were selected from districts in urban areas, and villages were selected from townships in rural areas; in the final stage, individuals resident in the selected areas were all invited to participate in the study.

A national survey had implied a hypertension prevalence in Chinese adults of 23.2% [11]. The estimation of the minimum required sample size was based on the following formula:

$$ \mathrm{N}={{\mathrm{Z}}_{\alpha}}^2\times p\ \left(1-p\right)/{d}^2 $$

Alpha (*α*) was the significance level; *p* was the prevalence of hypertension; *d* was the error tolerance which could be estimated as 0.10 × *p*. Z_*α*_ equals to 1.96 when *α* = 0.05*.* To reach a significance level of 0.05 and error tolerance of 0.10*p*, the estimated minimum sample size was 1272. Finally, a total of 4279 adults aged 20–80 participated in the survey.

### Measurements

Face to face questionnaire interviews, physical examinations were conducted by trained research staff. The following information was collected by questionnaire interview: demographic and socioeconomic data; personal history of hypertension; life style factors such as cigarette smoking, alcohol consumption and physical activities.

By using fixed stadiometer and body composition analyzer (BC-420, TANITA, Japan), we conducted anthropometric measurements to collect data on height and weight for the calculation of body mass index (BMI). Waist circumference (WC), hip circumference and neck circumference (NC) was measured using a plastic tape to a precision within 1 mm. The measure of NC was from the midway of the neck, between mid-cervical spine and mid-anterior neck. To avoid measurement bias, there was only one staff assigned to measure NC, WC and hip circumference all through the survey.

Three blood pressure (BP) readings were made by skilled staff using a digital BP measuring device (Omron HEM-907, Japan). Before the blood pressure measurement, all participants were required to have at least five minutes rest, avoiding exercise, drinking alcohol or tea, or smoking for at least 30 mins. We collected overnight fasting venous blood samples for the measurements of serum lipid and glucose by Chemistry Analyzer (ROCHE Cobas8000C701, USA) at the General Hospital of Chinese People’s Liberation Army (PLA). Serum lipid test items included total cholesterol (TC), triglycerides (TG), high-density lipoprotein cholesterol (HDL-C) and low-density lipoprotein cholesterol (LDL-C).

The definitions of cigarette smoking status, alcohol consumption and physical activities were descripted in our previous publications [13, 15, 16]. Overweight and obesity were defined as body mass index between 25.0 and 29.9 and of ≥30, respectively [17]. HTN was defined as an average SBP ≥ 140 mmHg or DBP ≥ 90 mmHg, or self-reported diagnosis of HTN [11]; pre-HTN was defined as blood pressure in the range of 120–139/80–89 mmHg [18] without being diagnosed as HTN before the survey, or taking any HTN medication.

### Statistical analyses

We performed the statistical analyses using SAS version 9.4 for windows (SAS Institute Inc., Cary, NC, USA).

Continuous variables were presented as mean with SD, and categorical data as number with percentage. A *p*-value < 0.05 (two-tailed) was considered statistically significant. Chi-square tests or Student’s t-test (or Wilcoxon sign test) were used to compare characteristics of participants in the analytic sample. The scatter plots fitted by penalized B-spline models were used to illustrate the relationship between NC and blood pressure (both SBP and DBP) in stratified BMI categories.

Two-way analysis of covariates was performed using general linear regression model (GLM) to identify the association between NC and blood pressure (SBP and DBP), and the adjusted means of blood pressure in NC quartile groups were calculated and compared. Logistic regression models were used to calculate the multi-variable adjusted prevalence of pre-HTN and HTN [19]. Multi-category logistic regression models were used to yield adjusted odds ratios (ORs) and 95% confidence intervals (CIs) of NC for pre-HTN and HTN, respectively. We further did stratified analyses by BMI classification to understand the independent association between NC and pre-HTN and HTN. The areas under the receiver operating characteristic (ROC) curve (AUC) were calculated by logistic regression models and were used as measurement of predicative value of body fat indexes in identifying the presence of HTN.

In the sensitivity analyses, different definition of overweight and obesity (24.0 and 27.9 as overweight, ≥ 28 as obesity) [20] was used to test the consistency of the results. Percentile regression models were used as replacement of GLM models to explore the association between NC and blood pressure (SBP and DBP).

## Results

### Participants characteristics

A total of 4279 participants were included with a mean age of (46.90 ± 13.80) years. The demographic, clinical and lifestyle characteristics of the subjects were summarized in Table 1. The average NC was (36.07 ± 3.18) cm, and the overall prevalence of pre-HTN and HTN were 11.05 and 23.63%, respectively. Male participants had higher means of body mass index, NC, SBP, DBP and higher prevalence of both pre-HTN and HTN (*p* < 0.001).

**Table 1:** Demographic and clinical characteristics of adults aged 20–80 years in Shaanxi Province, China, 2014

Characteristics	Male (*n* = 1733)		Female (*n* = 2546)		Total (*n* = 4279)	
Age, years (mean, SD)	47.64	14.01	46.39	13.64	46.90	13.80
Age groups* (n, %)						
20-	232	13.39	376	14.77	608	14.21
30-	291	16.79	459	18.03	750	17.53
40-	408	23.54	593	23.29	1001	23.39
50-	412	23.77	664	26.08	1076	25.15
60–80	390	22.50	454	17.83	844	19.72
Education attainment* (n, %)						
Elementary school or below	143	8.25	394	15.48	537	12.55
High school	1021	58.92	1375	54.01	2396	55.99
College or higher	569	32.83	776	30.48	1345	31.43
Urban resident	1088	62.78	1621	63.67	2709	63.31
Cigarette smoking* (n, %)						
Never smoking	584	33.70	2513	98.70	3097	72.38
Ever smoking	1149	66.30	33	1.30	1182	27.62
Alcohol consumption* (n, %)						
Quit drinking	97	5.60	6	0.24	103	2.41
Regular drinking	279	16.10	22	0.86	301	7.03
Occasionally drinking	584	33.70	119	4.67	703	16.43
Never drinking	773	44.60	2399	94.23	3172	74.13
Physical activity* (n, %)						
Inactive	242	13.96	447	17.56	689	16.10
Moderate	1169	67.46	1918	75.33	3087	72.14
Active	320	18.47	180	7.07	500	11.68
Family history of HTN* (n, %)	697	44.13	1123	44.13	1820	42.54
BMI*, kg/m^2^ (mean, SD)	24.13	3.29	23.38	3.63	23.68	3.52
WC*, cm (mean, SD)	88.19	9.93	82.04	10.54	84.53	10.73
WHR* (mean, SD)	0.90	0.07	0.87	0.07	0.88	0.07
FPG, mmol/L (mean, SD)	5.33	1.35	5.17	1.24	5.24	1.29
TC, mmol/L (mean, SD)	4.34	0.86	4.46	0.96	4.41	0.92
TG, mmol/L (mean, SD)	1.75	1.19	1.53	1.30	1.62	1.26
LDL-C, mmol/L (mean, SD)	2.64	1.76	2.62	1.15	2.63	1.43
HDL-C, mmol/L (mean, SD)	1.24	2.32	1.39	1.48	1.33	1.87
NC*, cm (mean, SD)	38.70	2.55	34.27	2.15	36.07	3.18
SBP* mmHg (mean, SD)	124.70	15.47	118.04	18.60	120.73	17.70
DBP*, mmHg (mean, SD)	78.14	10.80	73.30	10.61	75.26	10.95
Antihypertensive medication usage (n, %#)	235	50.43	329	60.37	564	55.79
Pre-HTN* (n, %)	259	14.95	214	8.41	473	11.05
HTN* (n, %)	466	26.89	545	21.41	1011	23.63

*p < 0.001 for the compassion between sex groups. Numbers may not sum to group totals due to missing values in some variables. *BMI* Body mass index, *WC* Waist circumference, *WHR* Waist-hip ratio, *HTN* Hypertension, *NC* Neck circumference, *SBP* Systolic blood pressure, *DBP* Diastolic blood pressure; # calculated as HTN patients who took antihypertensive medication divided by the overall number of HTN patients

### Relationships between NC and blood pressure

**Fig. 1:**
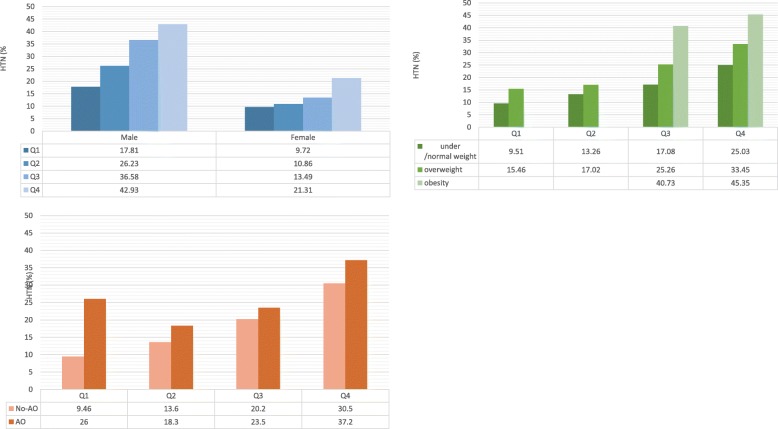
The adjusted prevalence of prehypertension and hypertension in adults aged 20–80 years in Shaanxi Province, China, 2014. Stratified by sex, neck circumference and body compositions. HTN: hypertension; Q1-Q4: the first to forth quartiles of neck circumference; AO: abdominal obesity

The associations between NC and blood pressure were presented in Fig. 1, where BMI categories (under/normal weight, overweight, obesity) were stratified to understand the independent association between NC and blood pressure. NC was observed significantly associated with increased SBP and DBP after adjusted for body composition indexes including WC, WHR and BMI (Additional file 1: Table S1, *p* < 0.001). In each BMI category, NC was also found positively associated with increased SBP and DBP (Additional file 1: Table S2, all *p* < 0.05). The scatter plots of NC and blood pressure fitted by penalized B-spline model revealed a positive association between NC and blood pressure (Additional file 1: Figure. S1).

### The association of NC with the prevalence and odds of pre-HTN and HTN

#### NC and pre-HTN/HTN prevalence

We calculated the adjusted prevalence of HTN in each BMI category across sex groups (Fig. 1). The adjusted covariates were age, educational attainment, residential areas (urban/rural), cigarette smoking status (ever smoke or never smoke). The prevalence of HTN increased with elevated NC quartiles in both sexes (*p* < 0.001). Male had higher prevalence of HTN than female within each NC quartile (*p* < 0.001). We further calculated the adjusted prevalence of HTN across BMI and NC categories (Fig. 1). The result indicated that the obese group with the fourth quartile of NC had the highest HTN prevalence (45.35%), and subjects of under/normal weight with the first quartile of NC had the lowest HTN prevalence (9.51%).

The stratified analyses in abdominal obesity status yielded similar results with BMI. People who were abdominal obese with the highest level of NC also had the highest adjusted prevalence of HTN (37.2%), compared with those in the same NC level but no-AO (30.5%) and other groups. Consistently, subjects who were no-AO and with the lowest level of NC had the lowest prevalence of HTN (9.46%) (Fig. 1).

### NC and the odds of pre-HTN and HTN

**Fig. 2:**
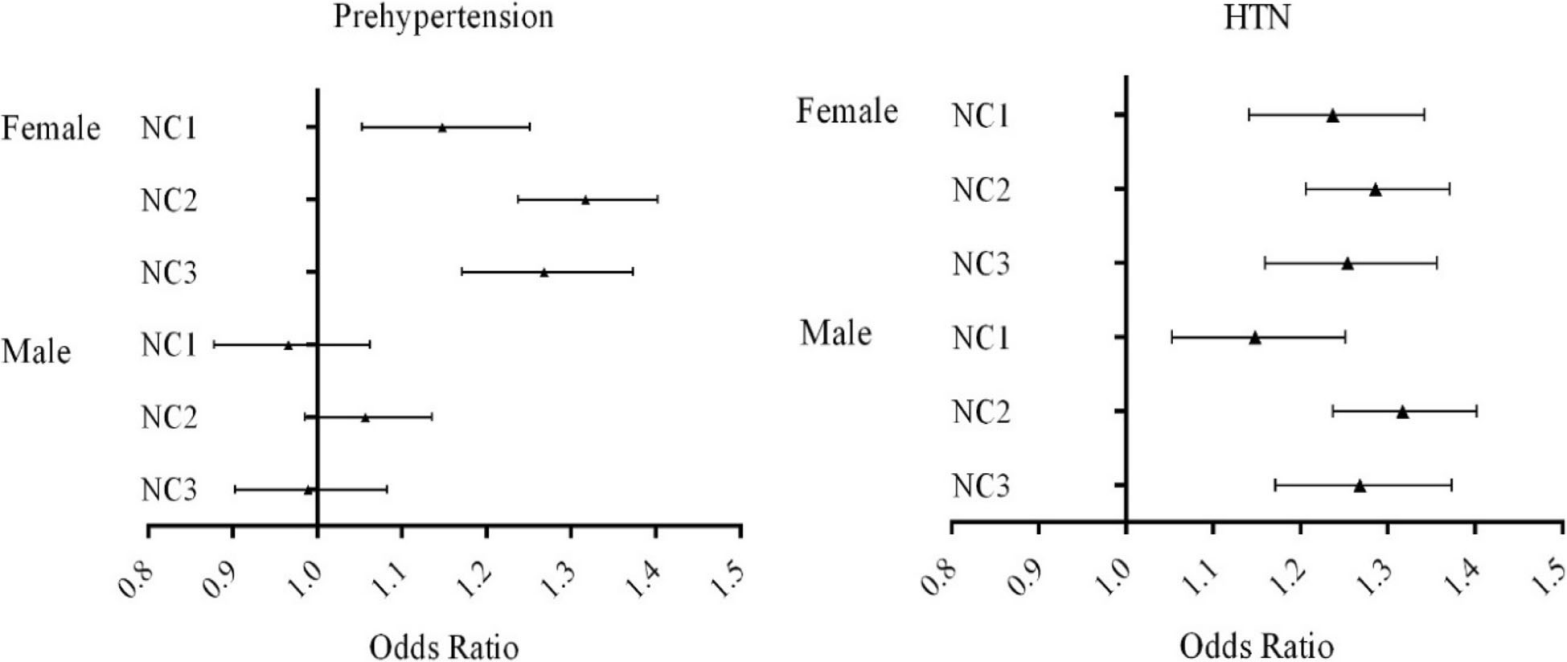
Forest plots based on multi-categorial logistic regression model illustrate the association between neck circumference and prehypertension/hypertension across sex groups in adults aged 20–80 years in Shaanxi Province, China, 2014. Adjusted covariates included sex, age, body composition, educational attainment, residential areas (urban/rural), smoking status (for males only), physical activity, serum lipids and fasting plasma glucose. NC: neck circumference; NC1: adjusted for body mass index; NC2: adjusted for waist-hip ratio; NC3: adjusted for waist circumference.

After adjusted for BMI, sex, life style factors (cigarette smoking, alcohol consumption, physical activity), family history of HTN and serum lipids (TC and LDL-C), NC was associated with HTN, with an OR of 1.22 (95%CI: 1.15–1.29), but no association was found between NC and pre-HTN (Fig. 2). Analyses stratified by sex indicated that, there were both significant associations between NC and pre-HTN and HTN in female (OR:1.17, 95%CI: 1.07–1.28), but no positive association between NC and pre-HTN in male (*p* = 0.210) (Table 2).

**Table 2:** the relationship of neck circumference with prehypertension and hypertension in Shaanxi adults aged 20–80 years in China, 2014 (*n* = 4279)

Pre-HTN	Model 1				Model 2			
	*OR*	*95%CI*		*p*	*OR*	*95%CI*		*p*
Overall	1.202	1.151	1.255	< 0.001	1.053	0.982	1.130	0.1475
Male	1.072	0.992	1.158	0.0792	1.052	0.972	1.140	0.2095
Female	1.150	1.052	1.256	0.0020	1.169	1.067	1.280	< 0.001
The effect of NC under different BMI categories								
Normal/under weight								
Overall	1.144	1.060	1.234	< 0.001	1.146	1.060	1.238	< 0.001
Male	1.099	0.993	1.216	0.0692	1.075	0.968	1.194	0.1740
Female	1.196	1.067	1.341	0.0021	1.210	1.077	1.361	0.0014
Overweight								
Overall	1.027	0.927	1.139	0.6099	1.034	0.930	1.149	0.5367
Male	0.985	0.863	1.124	0.8192	0.978	0.854	1.120	0.7484
Female	1.089	0.923	1.285	0.3136	1.115	0.942	1.319	0.2045
Obesity								
Overall	1.201	0.968	1.491	0.0960	1.224	0.960	1.561	0.1022
Male	1.332	0.967	1.836	0.0797	1.318	0.927	1.874	0.1246
Female	1.102	0.808	1.502	0.5399	1.196	0.830	1.724	0.3361
The effect of NC under abdominal obesity (AO) status								
AO								
Overall	1.099	0.985	1.227	0.0923	1.121	1.002	1.253	0.0462
Male	1.040	0.855	1.265	0.6944	1.070	0.862	1.329	0.5378
Female	1.127	0.986	1.287	0.0787	1.223	1.098	1.363	< 0.001
No-AO								
Overall	1.165	1.099	1.233	< 0.001	1.166	1.100	1.237	< 0.001
Male	1.138	1.063	1.219	< 0.001	1.127	1.051	1.210	< 0.001
Female	1.221	1.099	1.357	< 0.001	1.136	0.993	1.299	0.0634
HTN								
Overall	1.367	1.317	1.419	< 0.001	1.219	1.150	1.292	< 0.001
Male	1.279	1.192	1.371	< 0.001	1.258	1.171	1.353	< 0.001
Female	1.224	1.142	1.312	< 0.001	1.215	1.132	1.304	< 0.001
The effect of NC under different BMI categories								
Normal/under weight								
Overall	1.258	1.180	1.342	< 0.001	1.246	1.167	1.331	< 0.001
Male	1.302	1.186	1.429	< 0.001	1.281	1.164	1.410	< 0.001
Female	1.191	1.088	1.305	< 0.001	1.181	1.078	1.295	< 0.001
Overweight								
Overall	1.264	1.162	1.376	< 0.001	1.262	1.157	1.377	< 0.001
Male	1.233	1.098	1.384	< 0.001	1.233	1.094	1.389	< 0.001
Female	1.287	1.137	1.457	< 0.001	1.276	1.125	1.448	< 0.001
Obesity								
Overall	1.291	1.067	1.563	< 0.001	1.274	1.023	1.586	0.0304
Male	1.424	1.053	1.925	0.0216	1.481	1.056	2.075	0.0227
Female	1.203	0.936	1.546	0.1485	1.230	0.906	1.670	0.1852
The effect of NC under abdominal obesity (AO) status								
AO								
Overall	1.254	1.148	1.370	< 0.001	1.248	1.141	1.365	< 0.001
Male	1.237	1.012	1.513	0.0378	1.270	1.016	1.587	0.0357
Female	1.262	1.144	1.394	< 0.001	1.255	1.135	1.387	< 0.001
No-AO								
Overall	1.356	1.289	1.425	< 0.001	1.344	1.277	1.414	< 0.001
Male	1.372	1.289	1.460	< 0.001	1.354	1.271	1.444	< 0.001
Female	1.286	1.179	1.401	< 0.001	1.276	1.170	1.391	< 0.001

Model 1 adjusted for age, sex and BMI (categorized). Model 2: adjusted for age, sex (only in overall calculation), BMI, residential areas, education attainment, cigarette smoking (not in female), alcohol consumption (not in female), physical activity and serum lipids (TC and LDL-C). *BMI* Body mass index, *NC* Neck circumference, *HTN* Hypertension, *pre-HTN* Prehypertension, *AO* Abdominal obesity

Stratified analyses in BMI categories revealed that, in under/normal weight female, there was a positive association between NC and pre-HTN. No association was found in male sub-groups (all *p* > 0.10). For the odds of HTN, all sub-groups across sex and BMI categories, except for obese male, were observed having positive association between NC and the odds of HTN (Table 2). Stratified analyses in AO status indicated that, regardless of AO status, NC was found positively associated with HTN in both sexes. Whereas for pre-HTN, when only age was adjusted, NC was associated with pre-HTN in both AO and No-AO population in both sexes, but after multi-covariates were adjusted, the results seemed contradictory: NC was positively with pre-HTN in male AO subjects, but not for female, and in the No-AO population, the positive association was only observed in female participants.

To further understand the independent association between NC and elevated blood pressure, multiple body composition indexes such as WC and WHR were adjusted respectively in the logistic models (Fig. 2). The results showed that, there was sex difference in the association between NC and pre-HTN, which was stronger in female.

### The predictive value of NC compared with other body fat indexes

The areas under the ROC curves (AUCs) were constructed to evaluate the predictive value of NC and other body composition indexes for HTN (Table 3). There was no statistical difference of these predicative values (*p* > 0.05) with same sex and age-group. The AUCs of NC were 0.633 in male and 0.663 in female. BMI (0.669) and WC (0.716) had the highest values of AUCs in male and female, respectively. Sex and age-specified AUCs were calculated within each index. In male, NC had the highest predictive value in the 40–49 age group (0.714), and in female, the highest value fell in the 50–59 age group (0.658).

**Table 3:** Sex and age-specific areas under the receiver operating characteristic curves in the study population aged 20–80 years in Shaanxi Province, China, 2014

	NC			WC			WHR			BMI		
	AUC	95% CI		AUC	95% CI		AUC	95% CI		AUC	95% CI	
Male	0.633	0.604	0.662	0.662	0.634	0.690	0.655	0.627	0.683	0.669	0.641	0.696
20–39	0.662	0.585	0.739	0.669	0.597	0.742	0.643	0.570	0.716	0.715	0.644	0.787
40–49	0.714	0.655	0.774	0.665	0.602	0.727	0.624	0.560	0.688	0.686	0.627	0.744
50–59	0.668	0.614	0.722	0.658	0.604	0.713	0.634	0.578	0.690	0.685	0.632	0.738
60–80	0.667	0.613	0.721	0.663	0.610	0.717	0.635	0.580	0.690	0.675	0.622	0.728
Female	0.663	0.637	0.689	0.716	0.692	0.740	0.715	0.691	0.739	0.693	0.668	0.718
20–39	0.645	0.516	0.775	0.621	0.482	0.761	0.583	0.444	0.722	0.601	0.454	0.749
40–49	0.648	0.578	0.718	0.650	0.583	0.716	0.654	0.589	0.719	0.653	0.587	0.718
50–59	0.658	0.613	0.703	0.642	0.596	0.687	0.620	0.575	0.665	0.637	0.591	0.683
60–80	0.603	0.551	0.655	0.597	0.545	0.649	0.568	0.516	0.621	0.605	0.553	0.657

*AUC* Areas under the receiver operating characteristic curves, *NC* Neck circumference, *WC* Waist circumference, *WHR* waist-hip ratio, *BMI* Body mass index, *CI* confidence interval

## Discussion

Very few investigators have explored the potential value of NC measurements as an indicator of pre-HTN and HTN in general population in China. In this study, we found that NC was associated with blood pressure and both pre-HTN and HTN independently from BMI and other body fat indexes, in adults aged 20–80 years in Northwest China. After stratification analyses and regression analyses, we also found that the association varied within sex, age groups and BMI categories. The sensitivity analyses yielded similar results, which indicated that the study conclusions were robust.

Hypertension is a serious chronic health problem facing all age groups of population and has been aptly described as the top cause of death in China [21]. The staggering number of HTN in China (244.5 million) highlights the urgency to improve the capacity of primary healthcare services [10]. Regional deposition of fat, especially in the upper body segment, is a better predictor of some obesity related diseases including HTN [22]. As a portable and time saving anthropometric measurement, NC can be used in primary settings as an index of central obesity as well as in screening as a risk factor for HTN and other cardiometabolic diseases [6]. Comparing with other body composition indexes, such as BMI and Waist circumference (WC), NC measurement is more accurate: WC measurement may be time-consuming and culturally or environmentally problematic, especially in winter seasons due to thick clothes and affected by postprandial abdominal distension [22]; BMI could be fluctuating due to dietary or exercise factors much easier than NC, especially in young adults.

Previous studies have documented the value of NC as an indicator of HTN in Chinese population. In line with their findings [12, 23], increasing NC was observed associated with elevated SBP and DBP in both sexes, with stronger association with male blood pressure than that of female. Sex stratified analyses provided further information that the association of NC with pre-HTN has sex disparities, i.e. positive association was found only in female subjects. In contrast, Liang’s study [4] reported a disappeared association between NC and blood pressure and pre-HTN after the adjustment of BMI. This inconsistence may be attributable to its relatively smaller sample size (a total of 1709 participants), lack of information on sex stratification analysis and the heterogeneity of sample population (South China). The significance of the association between NC and pre-HTN/HTN were not changed after additional adjustment of BMI, although the magnitudes were slighted attenuated. However, when stratified by sex, the association lost its statistical significance in males, which was contrary with other studies [12, 24]. The inconsistencies between those prior studies and ours may be attribute to differences in genetic backgrounds, heterogeneity in sample population, or other confounding factors. In this study, the results suggested that BMI may play more important role on HTN risk in male than that in female.

The precise mechanisms underlying the associations between NC and blood pressure remain not fully understood. But in case of the upper half of body, the deposition of the adipose tissue in neck indicates the initial stage of obesity. Excess adiposity has been well studied as an important risk factors for cardiovascular risk factors, including hypertension [4–8]. NC could be demonstrated as predictive of future development of hypertension and more studies, especially prospective studies, are needed to further understand the mechanism of the association between NC and blood pressure.

We calculated AUCs to evaluate the predictive values of NC and other body composition indexes for HTN. According to the ROC analyses, however, NC may not be a good predictor of HTN due to its low value of AUC, that was 0.633 for male and 0.663 for female. There are few studies have compared the effect size of NC with WC, BMI, WHR or other body fat indexes in China. Zhou’s study [6] reported similar AUCs or NC to predict high BP in a Chinese population, but Fan’s study [12] observed a lower prediction value (AUC = 0.577 for female and 0.583 for male) in population in Southeast China. However, none of the studies provided data stratified by age groups, especially for younger people (20–35). Our study suggested that, in a relatively younger group (20–39), NC had lower predictive value (0.662 in male and 0.645 in female) in comparison with other body fat indexes in both sexes, which in male were 0.669 of WC, 0.643 of WHR, 0.669 of BMI, and in female were 0.621 of WC, 0.583 of WHR and 0.601 of BMI.

Certain limitations existed in this study and should be considered in interpreting the data. Firstly, the cross-sectional nature to some extend limits the ability to explore causal association between NC and blood pressure. Secondly, although we have carefully adjusted the possible confounders in the analyses, information on dietary intake, lifestyle situation was not yet complete. Thirdly, data analyses in this study were based on the study population, therefore it may not be appropriate to apply its conclusion to other population. Nonetheless, our data, derived from a representative population selected using multi-stage stratified cluster sampling method, could provide valuable information for practitioners, especially those in primary care settings, when it comes to screening for HTN.

## Conclusions

Our findings suggest that NC is a simple and useful anthropometric index to identify elevated blood pressure in adults of China. NC was associated with both pre-HTN and HTN independent from other body fat indexes.

## Supplementary information

**Additional file 1: Table S1**. Demographic and clinical characteristics of adults aged 20–80 years in Shaanxi Province, China, 2014. **Table S2.** The relationship between neck circumference and blood pressure, stratified by body composition categories, among Shaanxi adults aged 20–80 years in China, 2014 (*n* = 4279). **Figure. S1.** The scatter plots of neck circumference and blood pressure stratified by body weight in adults aged 20–80 years in Shaanxi Province, China, 2014. Fitted Penalized B-spline was used to yield the regression line and 95% confidence interval. (a): NC and SBP in normal/underweight group; (b): NC and SBP in overweight group; (c): NC and SBP in obese group; (d): NC and DBP in normal/underweight group; (e): NC and DBP in overweight group; (f): NC and DBP in obese group. NC: neck circumference. SBP: systolic blood pressure; DBP: diastolic blood pressure.

## Data Availability

The data that support the findings of this study are available from the Ministry of Science and Technology of China but restrictions apply to the availability of these data, which were used under license for the current study, and so are not publicly available. Data are however available from the authors upon reasonable request and with permission of the Ministry of Science and Technology of China.

## Abbreviations

AUC: Area under the receiver operating characteristic curve
BMI: Body mass index
BP: Blood pressure
CI: Conference interval
CNHS: China national health survey
DBP: Diastolic blood pressure
EBP: Elevated blood pressure
GLM: General linear regression model
HDL-C: High-density lipoprotein cholesterol
HTN: Hypertension
LDL-C: Low-density lipoprotein cholesterol
NC: Neck circumference
OR: Odds ratio
ROC: Receiver operating characteristic
SBP: Systolic blood pressure
TC: Total cholesterol
TG: Triglycerides
WC: Waist circumference
WHR: Waist-hip ratio
