# Supplementary material for: Neck circumference as an indicator of elevated blood pressure independent from body composition: implications from the China nation health survey (CNHS)
Source: BMC Cardiovasc Disord. 2019 Nov 6;19:244. doi: 10.1186/s12872-019-1227-8 (PMC6833177; doi:10.1186/s12872-019-1227-8)
Supplement: Supplementary file 1 — Additional file 1: Table S1. Demographic and clinical characteristics of adults aged 20–80 years in Shaanxi Province, China, 2014. Table S2. The relationship between neck circumference and blood pressure, stratified by body composition categories, among Shaanxi adults aged 20–80 years in China, 2014 (n = 4279). Figure. S1. The scatter plots of neck circumference and blood pressure stratified by body weight in adults aged 20–80 years in Shaanxi Province, China, 2014. Fitted Penalized B-spline was used to yield the regression line and 95% confidence interval. (a): NC and SBP in normal/underweight group; (b): NC and SBP in overweight group; (c): NC and SBP in obese group; (d): NC and DBP in normal/underweight group; (e): NC and DBP in overweight group; (f): NC and DBP in obese group. NC: neck circumference. SBP: systolic blood pressure; DBP: diastolic blood pressure. [file 12872_2019_1227_MOESM1_ESM.docx]

**Additional files**

**Table S1.** Demographic and clinical characteristics of adults aged 20-80 years in Shaanxi Province, China, 2014.

| Characteristics | Neck circumference | | | | | | | | Total (n=4,279) | |
| --- | --- | --- | --- | --- | --- | --- | --- | --- | --- | --- |
|  | Q1 (n=1073) | | Q2 (n=1053) | | Q3 (n=1083) | | Q4 (n=1070) | |  |  |
| Sex (n, %) |  |  |  |  |  |  |  |  |  |  |
| Male | 439 | 40.91 | 422 | 40.08 | 437 | 40.35 | 435 | 40.65 | 1733 | 40.5 |
| Female | 634 | 59.09 | 631 | 59.92 | 646 | 59.65 | 635 | 59.35 | 2546 | 59.5 |
| Age*, years (mean, SD) | 45.76 | 14.89 | 46.44 | 13.89 | 47.63 | 13.14 | 47.76 | 13.14 | 46.9 | 13.8 |
| Age group* 20- | 216 | 20.13 | 154 | 14.62 | 127 | 11.73 | 111 | 10.37 | 608 | 14.21 |
| 30- | 173 | 16.12 | 189 | 17.95 | 191 | 17.64 | 197 | 18.41 | 750 | 17.53 |
| 40- | 233 | 21.71 | 259 | 24.6 | 245 | 22.62 | 264 | 24.67 | 1001 | 23.39 |
| 50- | 244 | 22.74 | 260 | 24.69 | 302 | 27.89 | 270 | 25.23 | 1076 | 25.15 |
| 60-80 | 207 | 19.29 | 191 | 18.14 | 218 | 20.13 | 228 | 21.31 | 844 | 19.72 |
| Education attainment (n, %) |  |  |  |  |  |  |  |  |  |  |
| Elementary school or below | 113 | 10.53 | 126 | 11.97 | 140 | 12.93 | 158 | 14.77 | 537 | 12.55 |
| High school | 599 | 55.82 | 585 | 55.56 | 611 | 56.42 | 601 | 56.17 | 2396 | 55.99 |
| College or higher | 361 | 33.64 | 342 | 32.48 | 331 | 30.56 | 311 | 29.07 | 1345 | 31.43 |
| Urban resident | 693 | 64.59 | 663 | 62.96 | 698 | 64.45 | 655 | 61.21 | 2709 | 63.31 |
| Cigarette smoking (n, %) |  |  |  |  |  |  |  |  |  |  |
| Never smoking | 769 | 71.67 | 771 | 73.22 | 792 | 73.13 | 765 | 71.5 | 3,097 | 72.38 |
| Ever smoking | 304 | 28.33 | 282 | 26.78 | 291 | 26.87 | 305 | 28.5 | 1,182 | 27.62 |
| Alcohol consumption* (n, %) |  |  |  |  |  |  |  |  |  |  |
| Quit drinking | 23 | 2.14 | 24 | 2.28 | 34 | 3.14 | 22 | 2.06 | 103 | 2.41 |
| Regular drinking | 66 | 6.15 | 66 | 6.27 | 70 | 6.46 | 99 | 9.25 | 301 | 7.03 |
| Occasionally drinking | 171 | 15.94 | 177 | 16.81 | 180 | 16.62 | 175 | 16.36 | 703 | 16.43 |
| Never drinking | 813 | 75.77 | 786 | 74.64 | 799 | 73.78 | 774 | 72.34 | 3,172 | 74.13 |
| Physical activity (n, %) |  |  |  |  |  |  |  |  |  |  |
| Inactive | 172 | 16.03 | 169 | 16.05 | 169 | 15.6 | 179 | 16.73 | 689 | 16.1 |
| Moderate | 779 | 72.6 | 765 | 72.65 | 778 | 71.84 | 765 | 71.5 | 3,087 | 72.14 |
| Active | 120 | 11.18 | 119 | 11.3 | 135 | 12.47 | 126 | 11.78 | 500 | 11.68 |
| Family history of HTN* (n, %) | 401 | 37.37 | 443 | 42.07 | 461 | 42.57 | 515 | 48.13 | 1,820 | 42.54 |
| BMI*, kg/m2 (mean, SD) | 20.55 | 2.06 | 22.5 | 2.1 | 24.32 | 2.33 | 27.34 | 3.21 | 23.68 | 3.52 |
| WC*, cm (mean, SD) | 75.37 | 7.35 | 81.4 | 7.68 | 86.65 | 7.92 | 94.66 | 9.15 | 84.53 | 10.73 |
| WHR* (mean, SD) | 0.83 | 0.06 | 0.86 | 0.07 | 0.89 | 0.06 | 0.93 | 0.06 | 0.88 | 0.07 |
| FPG, mmol/L (mean, SD) | 5.04 | 1.18 | 5.2 | 1.39 | 5.25 | 1.21 | 5.46 | 1.33 | 5.24 | 1.29 |
| TC, mmol/L (mean, SD) | 4.28 | 0.9 | 4.4 | 0.98 | 4.43 | 0.88 | 4.54 | 0.9 | 4.41 | 0.92 |
| TG, mmol/L (mean, SD) | 1.22 | 0.78 | 1.46 | 0.95 | 1.71 | 1.15 | 2.08 | 1.77 | 1.62 | 1.26 |
| LDL-C, mmol/L (mean, SD) | 2.5 | 0.83 | 2.66 | 1.51 | 2.63 | 0.76 | 2.72 | 2.15 | 2.63 | 1.43 |
| HDL-C, mmol/L (mean, SD) | 1.47 | 1.82 | 1.31 | 0.33 | 1.25 | 0.64 | 1.3 | 3.17 | 1.33 | 1.87 |
| SBP*, mmHg (mean, SD) | 115.12 | 16.66 | 118.66 | 16.99 | 121.71 | 16.79 | 127.4 | 18.03 | 120.73 | 17.7 |
| DBP*, mmHg (mean, SD) | 71.54 | 10.14 | 73.77 | 9.77 | 75.97 | 10.43 | 79.72 | 11.67 | 75.26 | 10.95 |
| Antihypertensive medication usage* (n, %^#^) | 60 | 41.67 | 97 | 51.87 | 168 | 60.43 | 239 | 59.45 | 564 | 55.79 |
| Pre-HTN* (n, %) | 83 | 7.74 | 105 | 9.97 | 132 | 12.19 | 153 | 14.30 | 473 | 11.05 |
| HTN* (n, %) | 144 | 13.42 | 187 | 17.76 | 278 | 25.67 | 402 | 37.57 | 1,011 | 23.63 |

*p<0.05 for the compassion between neck circumference groups.  Numbers may not sum to group totals due to missing values in some variables. Q1-Q4: the first to forth quartiles of neck circumference. BMI: body mass index; WC: waist circumference; WHR: waist-hip ratio; HTN: hypertension; NC: neck circumference; SBP: systolic blood pressure; DBP: diastolic blood pressure; # calculated as HTN patients who took antihypertensive medication divided by the overall number of HTN patients.

**Table S2.** The relationship between neck circumference and blood pressure, stratified by body composition categories, among Shaanxi adults, aged 20-80 years in China, 2014 (n=3,714) ^a^.

|  | SBP (mmHg) | | | | | DBP (mmHg) | | | | |
| --- | --- | --- | --- | --- | --- | --- | --- | --- | --- | --- |
|  | *B* | *SE* | *95%CI* | | *P* | *B* | *SE* | *95%CI* | | *p* |
| NC1 ^b^ | 0.853 | 0.153 | 0.552 | 1.153 | <0.001 | 0.444 | 0.106 | 0.237 | 0.651 | <0.001 |
| NC2 | 1.172 | 0.117 | 0.943 | 1.402 | <0.001 | 0.867 | 0.081 | 0.708 | 1.026 | <0.001 |
| NC3 | 0.997 | 0.147 | 0.708 | 1.286 | <0.001 | 0.582 | 0.102 | 0.383 | 0.781 | <0.001 |
| The effect of NC under different body mass index categories ^b^ | | | | | | | |  |  |  |
| Normal/underweight | 1.069 | 0.148 | 0.779 | 1.359 | <0.001 | 0.817 | 0.104 | 0.613 | 1.022 | <0.001 |
| Overweight | 1.407 | 0.282 | 0.854 | 1.959 | <0.001 | 0.817 | 0.180 | 0.464 | 1.169 | <0.001 |
| Obesity | 1.276 | 0.577 | 0.145 | 2.407 | <0.001 | 0.739 | 0.371 | 0.011 | 1.467 | 0.047 |
| The effect of NC under abdominal obesity (AO) status ^c^ | | | | | | |  |  |  |  |
| AO | 1.275 | 0.311 | 0.666 | 1.884 | <0.001 | 0.755 | 0.207 | 0.349 | 1.160 | <0.001 |
| No-AO | 1.418 | 0.122 | 1.179 | 1.657 | <0.001 | 1.020 | 0.085 | 0.853 | 1.186 | <0.001 |

a: the sample was restricted to participants without taking antihypertensive medications.

b:NC1: general linear models were performed with covariates adjusted including BMI, sex, age, residential areas, educational attainment, cigarette smoking, alcohol consumption and physical activity. NC2: adjusted for waist-hip ratio instead of BMI based on NC1; NC3: adjusted for waist circumference instead of BMI based on NC1. b: general linear models were used within each body mass index category, the adjusted covariates included sex, age, residential areas, educational attainment, cigarette smoking, alcohol consumption and physical activity; c: general linear models were used within each abdominal obesity category, the adjusted covariates included sex, age, residential areas, educational attainment, cigarette smoking, alcohol consumption and physical activity. B: regression coefficient; SE: the standard error of the regression coefficient.

(b)

**Figure S1.** The scatter plots of neck circumference and blood pressure stratified by body weight in adults without taking antihypertensive medications, aged 20-80 years in Shaanxi Province, China, 2014. Fitted Penalized B-spline was used to yield the regression line and 95% confidence interval. (a): NC and SBP in normal/underweight group; (b): NC and SBP in overweight group; (c): NC and SBP in obese group; (d): NC and DBP in normal/underweight group; (e): NC and DBP in overweight group; (f): NC and DBP in obese group. NC: neck circumference. SBP: systolic blood pressure; DBP: diastolic blood pressure.


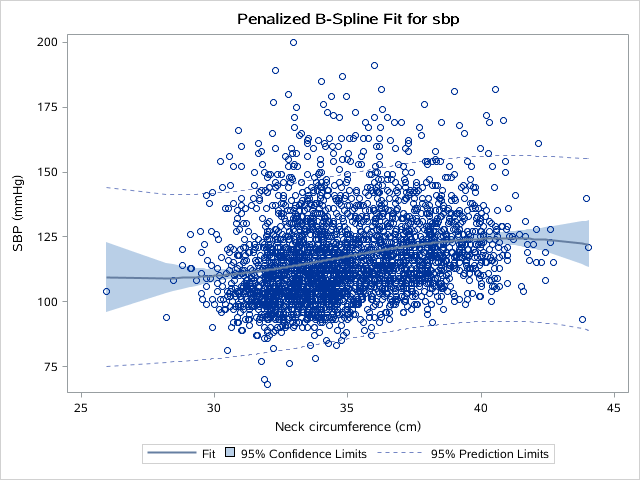

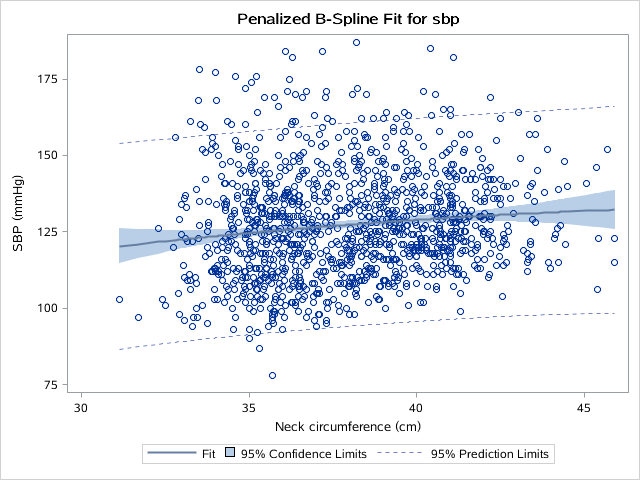

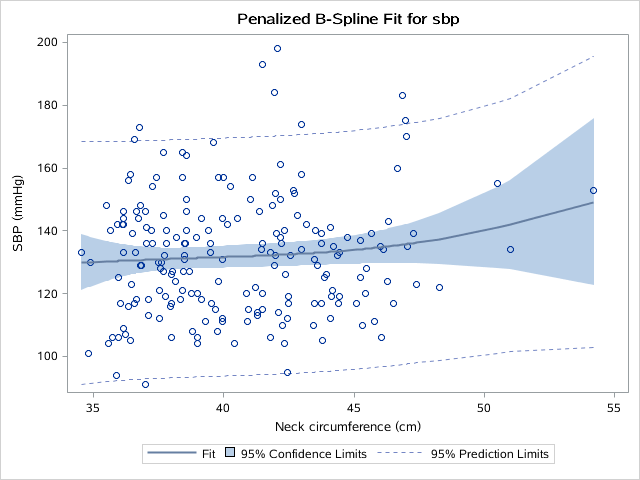


(c)


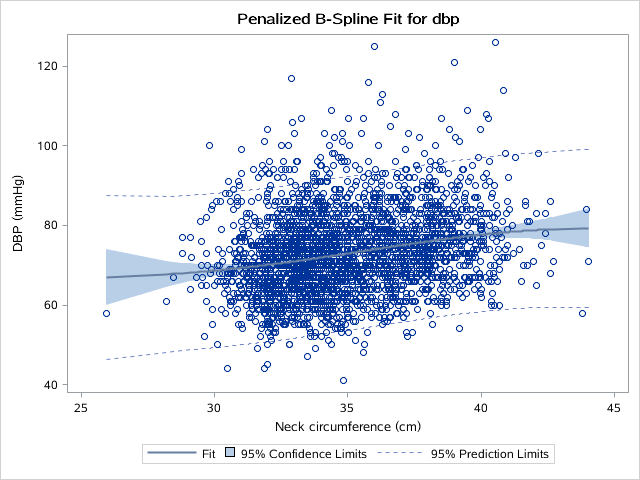

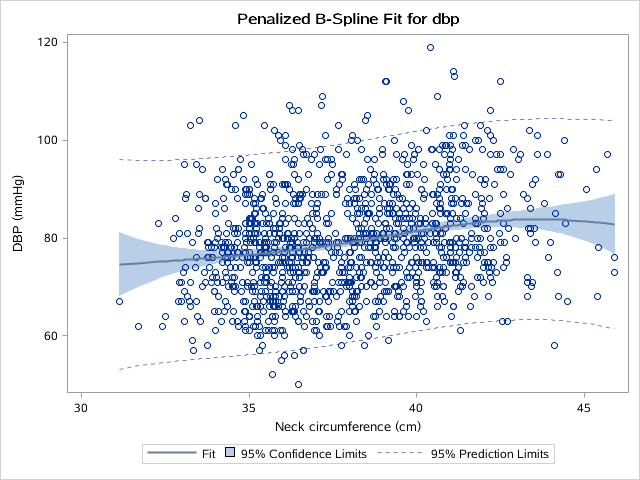

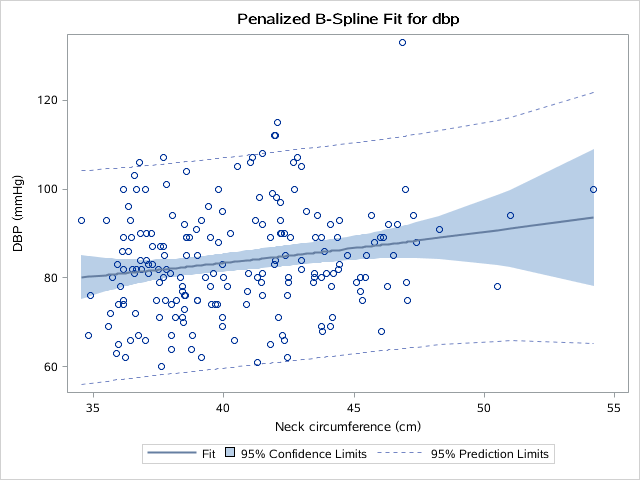


(d)

(e)

(f)

(a)
